# “CLOVER” Model of Service and Training Development for the Well‐being of People with Dementia in Residential Care Homes of Hong Kong

**DOI:** 10.1002/alz70858_097640

**Published:** 2025-12-24

**Authors:** Kenny Chi Man Chui

**Affiliations:** ^1^ Jockey Club Centre for Positive Ageing, The Chinese University of Hong Kong, Hong Kong, Hong Kong

## Abstract

In the context of Hong Kong, the ageing population becomes one of the factors that increase the number of people with dementia, and, surprisingly, having dementia also becomes one of the reasons that the care partners will send the person to residential care services with too early institutionalization. From the local research, around 70% of the residents living in the residential care homes had different levels of cognitive decline or dementia and worse, some of the residents did not have a proper diagnosis. The diagnostic rate of dementia is around 15%, which is relatively low, and senior citizens are required to wait for over 100 weeks to have the proper medical review. The use of restraints for people with dementia in the residential care homes of Hong Kong was easy to find. There is an urge to create a culturally sensitive model of dementia care practice with the adaptation of person‐centred care (PCC) in residential care homes in Hong Kong.

With the application of a qualitative research approach from participatory observation and in‐depth interviews, we learn from the voices of people with dementia and their care partners, as well as the caring staff of over 20 residential care homes in Hong Kong. Thematic analysis and literature reviews guide the development of the “CLOVER” model to facilitate the implementation of person‐centred care in residential care homes. It also helps to provide a restraint‐free environment to preserve the essential quality of life and dignity.

The “CLOVER” model stands for “C”‐Care with PCC theory; “L”‐Love with Kitwood's flower; “O”‐Observe with PCC communication regarding dementia care mapping; “V”‐Voice with 16 principles of proper caring attitude from the voice of people with dementia (DemenTitude®) in Hong Kong; “E”‐Evidence‐based practice with research and evaluation; “R”‐Reablement with dementia inclusive environment and meaningful engagement.

The “CLOVER” model provides six core elements as the references for implementing holistic care services and the structure of staff training so that the well‐being of residents with dementia can be upheld. With another meaning of “clover” (Trifolium), everyone should present “hope, faith and love” with luck to people with dementia.

